# Erythrocyte Load in Cerebrospinal Fluid Linked with Hippocampal Atrophy in Alzheimer’s Disease

**DOI:** 10.3390/jcm14134670

**Published:** 2025-07-01

**Authors:** Rafail Christodoulou, Georgios Vamvouras, Laura Lorentzen, Evros Vassiliou

**Affiliations:** 1 Department of Radiology, Stanford University School of Medicine, Stanford, CA 94305, USA; 2 Department of Mechanical Engineering, National Technical University of Athens, 157 72 Zografou, Greece; 3 Department of Biological Sciences, Kean University, Union, NJ 07083, USA; llorentz@kean.edu (L.L.); evassili@kean.edu (E.V.)

**Keywords:** hippocampal volume, Alzheimer’s disease, erythrocytes, cerebrospinal fluid

## Abstract

**Background:** Alzheimer’s disease is a neurodegenerative disease of unknown etiology. Employing a combination of techniques such as imaging modalities, cognitive tests and medical history evaluations is considered to be a reliable approach in diagnosing the disease. A characteristic feature of Alzheimer’s disease is the gradual atrophy of the hippocampus, which is also seen with aging but at a faster rate in individuals suffering from the disease. The trigger responsible for the atrophy remains unknown. **Methods:** In this study, patients were assessed using MRI brain imaging, blood and cerebrospinal fluid analysis. **Results:** The findings indicate that the levels of erythrocytes in the cerebrospinal fluid have a statistically significant longitudinal predictive marker effect on hippocampal atrophy. Mean arterial pressure showed modest statistical significance in hippocampal volume only in the Alzheimer’s disease group. **Conclusions:** The results of the study point to the significance of cerebrospinal fluid homeostasis in terms of elements capable of causing hippocampal atrophy under chronic conditions. Monitoring of the presence of erythrocytes in cerebrospinal fluid and their related metabolites may be of clinical significance in the long-term management of Alzheimer’s disease.

## 1. Introduction

Alzheimer’s disease (AD) is a neurodegenerative disease of unknown causes where aging is considered a major risk factor [1,2]. It is the most common cause of dementia characterized by gradual neuronal death and progressive memory loss along with a reduction in complex cognitive skills [3,4]. The degeneration of the neurons leads to a reduction in hippocampal volume [5,6]. While levels of phosphorylated tau (p-tau) both in the blood and brain positively correlate with AD, the actual cause of the disease remains unclear [7,8]. In addition to p-tau, beta amyloid proteins in the blood, particularly the ratio of beta amyloid 42 to beta amyloid 40 (Aβ42/Aβ40), also correlates with an increased risk of dementia and AD progression [9,10,11].

An area of intense scientific interest is the role of the blood brain barrier (BBB) with respect to AD [12,13,14]. The BBB is a complex filter that prevents toxic substances in the blood from reaching the brain while facilitating the removal of waste substances generated from the brain [15,16]. This process is accomplished by a variety of cell types, such as endothelial cells, pericyte cells and astrocyte end-feet. Micro-hemorrhaging into the cerebrospinal fluid (CSF) introduces erythrocytes in the CSF that require proper recycling to avoid toxicity [17,18]. In the central nervous system (CNS), the removal of erythrocytes is primarily carried out by microglia, which is a process known as erythrophagocytosis [19]. Upon red blood cell engulfment, microglia enzymatically break down heme using the heme oxygenase (HO) enzyme [20,21]. The byproducts of heme metabolism (carbon monoxide gas, bilirubin and ferrous ion) provide immunomodulation and neuroprotection [22]. High activity of HO has been observed in the spleen where the bulk of erythrocyte recycling takes place [23]. In the CNS, though, this responsibility is primarily undertaken by microglia. It is rather interesting that carbon monoxide, a toxic gas, provides neuroprotection upon traumatic brain injury typically associated with hemorrhaging [24]. It must be noted, though, that this phenomenon is a response to stress due to the presence of erythrocytes in the CSF. Chronic exposure to erythrocytes and their subsequent elimination via microglial engulfment is very likely to be toxic to the brain. An alternative mechanism through which erythrocyte elimination can occur is via the arachnoid granules [25]. This pathway is negatively impacted by erythrocytes and related byproducts of heme metabolism. Their presence interferes with normal CSF flow and blood clearance [26]. Moreover, the permeability of arachnoid membranes is distorted, which can potentially affect the survival of neurons. An alternative route of blood-derived waste product removal in the CSF is via meningeal lymphatic drainage [27]. In mice, administration of endothelial factor C increased meningeal lymphatic drainage [28]. The increased drainage of macromolecules in the CSF led to an increase in both memory performance and learning. Even more relevant to AD was the observation that in transgenic models of AD, the disruption of meningeal lymphatic drainage led to β amyloid deposition in the meninges, mimicking human AD pathology.

In cases where erythrocytes enter the CSF due to hemorrhaging because of cerebral vessel rupture, specialized proteins, such as haptoglobin, exhibit high affinity for hemoglobin, acting as a buffer and provide protection against hemoglobin toxicity [29]. Similar to haptoglobin, hemopexin has high affinity for heme, which is a critical component of hemoglobin that is responsible for oxygen binding [30,31]. Overexpression of hemopexin leads to improved outcomes with respect to brain tissue injury, peroxidation, hematoma volumes and lesion size. Low CSF levels of hemopexin are associated with increased AD pathology, low hippocampal metabolism and mental deterioration [32].

An additional mechanism of CSF hemoglobin toxicity is interference in cerebral blood flow. Blood oxygenation level-dependent cerebrovascular reactivity (BOLD-CVR) whole brain imaging showed that the infusion of hemoglobin in CSF led to rapid cerebrovascular reactivity impairment [33]. Furthermore, haptoglobin co-infusion with hemoglobin attenuated cerebrovascular impairment in sheep [34].

Several routes through which waste products are eliminated ensure that the CSF is devoid of toxic agents that can harm the brain. In cases where the BBB becomes compromised either due to age or trauma, the burden of waste removal increases dramatically and leads to saturation. The long-term accumulation of erythrocytes in the CSF is associated with considerable toxicity to the entire brain.

Given this observation, this study chose to analyze two relevant parameters. First, the levels of erythrocytes in CSF (CTRED) and, second, the mean arterial pressure (MAPres). It was hypothesized that high arterial pressure may be associated with increased erythrocyte leakage via the BBB into the CSF. Consequently, the chronic presence of erythrocytes in the CSF, whether a byproduct of high arterial pressure or a deterioration of the BBB, may be playing a role in hippocampal atrophy.

## 2. Results

### 2.1. MRI Imaging of the Hippocampus of a Control Patient vs. An Alzheimer’s Disease Patient

**Figure 1:**
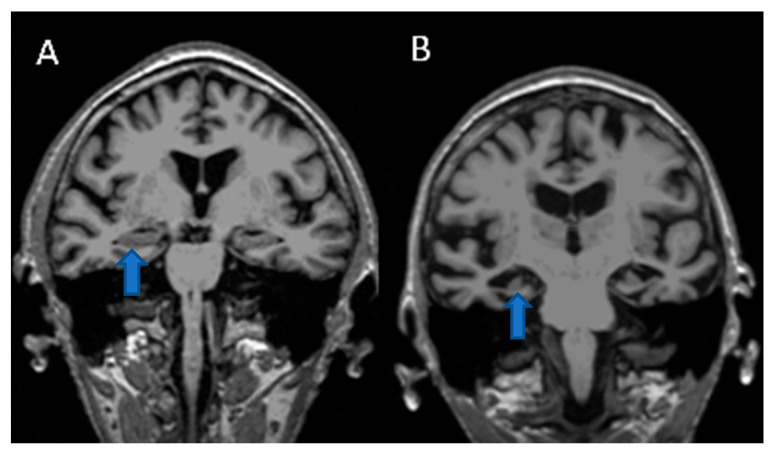
MRI brain images (blue arrows point to hippocampus) from (**A**) a control patient and (**B**) an Alzheimer’s disease patient.

MRI imaging is frequently used to assess hippocampal volume. In Figure 1, a comparison of a control patient (A) is shown in comparison to an AD patient (B). As seen from the MRI images, there is a noticeable difference in hippocampal volume between the control patient and the AD patient.

### 2.2. Hippocampal Volume Controls vs. Alzheimer’s Disease

**Figure 2:**
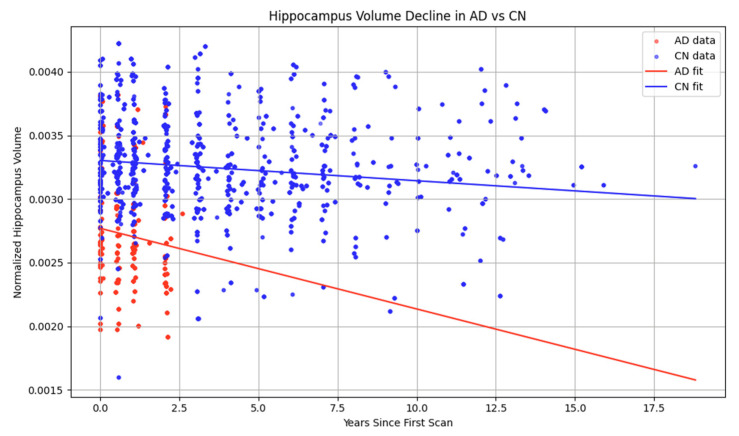
Hippocampal volume of cognitively normal subjects (CN) and Alzheimer’s disease (AD) subjects.

A known characteristic of AD is the reduction in hippocampal volume in parallel to the progression of the disease [5,35]. Unsurprisingly, the normalized baseline volume of cognitively normal subjects (CN) was higher compared to AD subjects (0.002766). The difference (−0.000533) was statistically significant (Figure 2, Table 1). The rate of decline in AD (−0.000071 per year) was approximately 4x faster in comparison to CN (−0.000016 per year). The additional decline in AD (−0.000047/year) was statistically significant. As expected, the model robustly supports that AD is associated with both lower starting hippocampal volume and faster atrophy over time. The high statistical significance across all terms strengthens the evidence for distinct neurodegenerative trajectories between CN and AD. It should be noted that the magnitude of the coefficients is very small due to the unit scale of the variables, and this does not directly reflect the strength of the association, which is better captured by the relative changes or standardized effect sizes (i.e., correlation coefficients).

### 2.3. CSF Erythrocyte Load vs. Hippocampal Volume

The cerebrospinal erythrocyte load in the CSF was assessed by examining the erythrocyte count in the CSF (CTRED). An examination of the simple correlation between CTRED and hippocampal volume exhibited the following:

- All subjects: *r* = *0.049*, *p* = *0.0466*.
- AD: *r* = −*0.021*, *p* = *0.662*.
- CN: *r* = +*0.076*, *p* = *0.0071*.

This result indicates that while the global correlation is weakly positive and barely significant, the group-wise results showed no association in AD and a slight positive trend in CN subjects.

### 2.4. Mean Arterial Pressure (MAPres) vs. Hippocampal Volume

An examination of the MAPres vs. hippocampal volume cross-sectional analysis exhibited the following:

– All subjects: *r* = −*0.024*, *p* = *0.157*.
– AD: *r* = +*0.096*, *p* = *0.004*.
– CN: *r* = −*0.029*, *p* = *0.130*.

**Figure 3:**
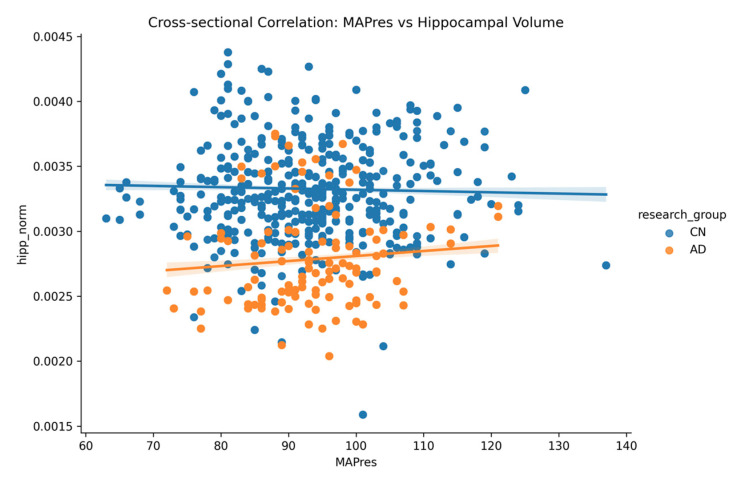
Correlation of mean arterial pressure (MAPres) and hippocampal volume in cognitively normal (CN) and Alzheimer’s disease (AD) subjects.

A statistically significant positive correlation was observed only in the AD group (*r* = +0.096, *p* = 0.004), suggesting that higher MAPres values may be modestly associated with greater hippocampal volume in individuals with AD (Figure 3). In contrast, no significant relationship was found in cognitively normal (CN) subjects (*r* = −0.029, *p* = 0.130) or in the overall subject group (*r* = −0.024, *p* = 0.157), indicating that the observed effect is specific to the AD population and is not a general trend across all subjects.

### 2.5. Longitudinal Mixed-Effects Model (Lagged Predictors)

Mixed-effects models were fitted to assess whether prior values of CTRED and MAPres could predict subsequent hippocampal volume loss.

**Model 1:** *hipp_norm* ~ *z_prev_CTRED* + YearsSinceFirst * research_group

This translates to the following mathematical formula:

*hipp_norm* = *β_0* + *β_1∙z_prev_CTRED* + *β_2∙YearsSinceFirst* + *β_3∙research_group* + *β_4∙(YearsSinceFirst* × *research_group)* + *random effects*

The results of the mixed-effects model suggest that higher CTRED values at the previous time-point are associated with lower hippocampal volume at the subsequent visit (Table 2). This association is statistically significant despite the small coefficient, indicating that CTRED may carry predictive information about future neurodegeneration.

**Model 2:** *hipp_norm* ~ *z_prev_MAPres* + YearsSinceFirst * research_group

This formula is expanded as follows:

hipp_norm = β_0_ + β_1_ • z_prev_MAPres + β_2_ • YearsSinceFirst + β_3_ • research_group + β_4_ • (YearsSinceFirst × research_group) + random effects

In contrast, when MAPres is used as the predictor, the model did not find a statistically significant relationship between prior MAPres values and future hippocampal volume (Table 3). Although MAPres showed some interesting correlations in the cross-sectional AD group, it did not retain predictive power in this longitudinal setting. This finding indicates that MAPres may reflect a concurrent physiological state rather than forecast future structural changes in the brain. The time and group effects remain consistent with Model 1. That is, hippocampal volume declines over time, with CN subjects showing a slower rate of decline, and CN individuals starting from a higher baseline hippocampal volume. These patterns reinforce the robustness of group and time effects independently of the specific physiological predictor.

## 3. Discussion

Unsurprisingly, there are some limitations in the study. The number of subjects in the AD group was substantially smaller than the control. Furthermore, the time range for the AD group was smaller than the CN group. Both impediments can conceivably be addressed by revisiting the database at a future point and repeating the analysis with a larger patient pool. The ADNI 4 data collection is scheduled to continue until 2027. Some imaging data was of lower quality, which may have affected the correlation values due to noise affecting volume measurements. The current imaging data is typically of higher quality in comparison to more dated scans. Again, a future data analysis will remedy this issue as more scans with higher resolution will be acquired. Another technical constraint is the invasive nature of CSF acquisition and erythrocyte measurement. Currently, there is no imaging technique to assess erythrocyte or hemoglobin levels in CSF in a non-invasive manner. The recent FDA approval of a blood assay to assess beta amyloid and tau levels in plasma for the purposes of AD diagnosis will likely result in even fewer patient cases with direct CSF analysis. In the future, the possibility of the development of an imaging technique to assess hemoglobin levels in CSF cannot be ruled out. Such technology would allow for frequent monitoring and would shed more light on the long-term impact of erythrocytes and hemoglobin levels on hippocampal atrophy.

Nonetheless, the findings derived from Model 1 (CTRED predictor) and Model 2 (MAPres predictor) support the hypothesis that increased cerebrospinal erythrocyte load measured as CTRED is linked with ongoing hippocampal atrophy in AD. As expected, hippocampal volume decreased over time, reflecting the progressive nature of neurodegeneration. This time effect was especially pronounced in the AD group, which is consistent with established disease trajectories. Importantly, Model 1 included an interaction term between time and diagnostic group. This interaction showed that the rate of volume loss was significantly slower in cognitively normal (CN) individuals compared to the AD group. In other words, while both groups experience some decline in hippocampal volume with age, CN subjects decline more gradually. Also, the model suggests that although the baseline volume may not differ much, CN individuals maintain higher hippocampal integrity in the long run. Overall, the erythrocyte load in CSF showed a statistically significant negative association with future hippocampal volume. This finding is relevant in the AD group where brain atrophy was more pronounced. Contrary to CTRED, mean arterial pressure (MAPres), while significant in cross-sectional AD analysis, did not hold predictive power longitudinally. Blood pressure medications (beta-blockers) that are capable of crossing the BBB have been linked to a lower risk for AD [36]. Their therapeutic benefit is thought to be related to increased waste brain metabolite clearance. The chronic presence of erythrocytes in the CSF will eventually lead to waste products that are likely harmful to the brain and hippocampus. Similar toxic findings from erythrocytes and subsequent hemoglobin release have been reported in patients who experienced an aneurysmal subarachnoid hemorrhage [37]. Treatment with haptoglobin and hemopexin reduced both the lipid peroxidation and vasoconstrictive effects of aneurysmal hemorrhaging. Bi-daily intracerebroventricular injections of hemoglobin for merely three days were sufficient to negatively affect food intake, movement and neurological scores in sheep [34]. The neurotoxic effects of hemoglobin were substantially reduced when equimolar amounts of haptoglobin and hemoglobin were administered.

Cerebral vascular angiopathy (CSA) and AD are intertwined with beta amyloid deposition [38]. It should not be surprising that micro-hemorrhaging through cerebral vasculature can lead to increased erythrocyte load in CSF. It has recently been hypothesized that increased clearance of amyloid beta in CSF could be an effective strategy in the treatment of AD [39]. The so-called “cerebrospinal fluid sink therapeutic strategy” in the context of beta amyloid may also be applied to erythrocyte metabolite waste products. The experimental use of implantable devices for clearing waste products is currently under investigation and may prove to be a valuable tool in the management of AD [40]. Bedside proof-of-principle devices for monitoring levels of hemoglobin in CSF, especially in the event of aneurysmal hemorrhaging, highlight the clinical significance of hemoglobin within the frame of neurotoxicity [41].

## 4. Materials and Method

The tabular data of the patient characteristics (Table 4), as well as the corresponding images, were obtained from the ADNI database (https://adni.loni.usc.edu/, access date: 9 March 2025). Since the study focuses on AD and not on other types or stages of dementia, only subjects labeled as cognitively normal (CN) or Alzheimer’s disease (AD) were sampled, excluding various other categories, such as mildly cognitively impaired (MCI). This decision was made to ensure clarity in the classification and avoid overlap between transitional stages that could dampen the statistical relationships under investigation. After filtering for visits in which subjects had acquired both T1-weighted MRI and non-imaging examinations, the total number of usable visits was 2885. In addition, case selection was carried out in two phases. Initially, only the subjects with at least two visits that included both the MAPres and CTRED measurements, as well as an MRI scan with temporal proximity to the aforementioned measurements, were included. Subjects with only one such visit were excluded since the analysis is longitudinal. The second screening was based on the type of MRI for each subject and visit. Localizer, field mapping, Gradwarp, scaled scans and N3 scans were omitted because they are either incompatible with FreeSurfer or do not include the necessary information for segmentation, or both.

The classification of subjects in the ADNI database was conducted based on clinical features (patient history), imaging findings (MRI and PET), neuropsychiatric cognitive tests (MMSE) and biomarkers. Clinicians took multiple parameters into consideration to diagnose and label subjects as AD or cognitively normal (CN). In terms of variable selection, multiple variables were extracted from the ADNI database. Upon analysis, the majority of these variables had no clinical or statistical significance. MAPres and CSF erythrocyte levels were chosen due to their statistical significance in terms of hippocampal atrophy and relevance to AD progression.

The images were obtained in their raw form as DICOM files. A processing pipeline was used to extract the normalized left and right hippocampal volumes. Segmentation was performed using the openly available FreeSurfer software (https://surfer.nmr.mgh.harvard.edu/, v.7.4.1), which automatically computed the necessary volume values, among other information such as full brain volume that was used for normalization. This approach has been extensively validated in the literature and remains a standard tool in neuroimaging studies of brain atrophy. Since each scan needed 6 to 8 h to be fully processed by FreeSurfer, a method was devised for optimal use of available computational resources with the aim of maximizing speed and efficiency. The process was run in a Google Cloud Platform (GCP) Virtual Machine (VM) of N2-standard architecture which is based on Intel^®^ Xeon^®^ Cascade Lake processors and included 8 vCPUs and 32 GB RAM. This meant that 8 threads were available, and thus 8 processes could run in parallel, at any given moment in time. Using cloud computing proved highly effective, as it allowed scaling up of the analysis without the constraints imposed by local hardware limitations. The pipeline, which automated the entire process using a collection of shell scripts, thus enabling unattended execution, is summarized below:

1. Unzip DICOM data.
2. Convert DICOM data to NIFTI.
3. Until all scans are processed, do the following:
  - Load 8 scans in parallel.
  - Observe which of the three following events occurs:
    - An error occurs during processing of a scan. (Errors could be caused by corrupt scans, very low resolution, extreme artifacts, and heavy editing from the source, as well as the depiction of unrelated areas of the brain instead of a full brain scan (which is eligible for use in FreeSurfer)).
    - A scan has been successfully segmented.
    - A scan type is unusable for segmentation analysis.
    Then, catalog the event of success, failure or omission, save the available results, and replace it with a new one from the heap of unprocessed scans, so that at every moment, the maximum number of scans is being processed and no processor is left idle.

After all scans had been segmented, a secondary extraction step was performed to collect left and right hippocampal volumes from the FreeSurfer logging files and normalize the values using the full brain volume. This step ensured inter-subject comparability by taking total brain size variability into account, which is known to influence volumetric interpretations. The normalized values were then appended to the respective subject and visit entry in a central CSV file for further statistical analysis.

It should be noted that scans labeled by ADNI as “GradWarp”, “Scaled”, “Localizer”, “Field Mapping” and “N3” were automatically omitted as they do not contain the necessary information for segmentation and volume extraction. Also, the MRI acquisition dates almost never matched the visit to the day, so a margin was allowed in the matching process, which could not exceed three months.

Statistical analysis then followed, after screening for outliers (unnatural or extreme volume values) and keeping subjects that had at least two visits, so that temporal relationships could be recorded. As mentioned before, hippocampal volume was averaged between the two left and right hippocampi, and normalized by the full brain volume, as shown in the following equation:

$$\mathrm{hipp}\_\mathrm{norm}=\frac{\mathrm{left}\;\mathrm{hippocampus}+\mathrm{right}\;\mathrm{hippocampus}}{2\times \mathrm{total}\;\mathrm{brain}\;\mathrm{volume}}$$

Cross-sectional correlation relationships between each of the mentioned biomarkers (CTRED, CTWHITE, PROTEIN and MAPres) and the normalized hippocampal volume were assessed using Pearson correlation coefficients across the full data cohort and for each separate patient group (AD vs. CN). This approach allowed for the investigation of global as well as group-specific associations.

Additionally, separate linear mixed-effects models were fitted for each biomarker. Specifically, the dependent variable (hipp_norm = normalized hippocampal volume) was expressed as a linear function of the main predictor (z_prec_*BIOMARKER* = lagged z-scored biomarker), the covariates (YearsSinceFirstScan, research_group) and their interaction, and random effects in order to account for the noise. The model structure, as mentioned previously, is:

hipp_norm ~ z_prev_*BIOMARKER* + YearsSinceFirst × research_group

Hippocampal volume values below the 3rd percentile and above the 97th percentile were excluded as outliers, after having checked that the said values were physiologically implausible, to ensure stability in model fitting and avoid the influence of artifacts or segmentation errors.

## Figures and Tables

**Table 1:** Statistical analysis of hippocampal volume decline.

Predictor	Coef.	*p*-Value
Intercept	0.003303	<0.001
research_group[T.AD]	−0.000533	<0.001
YearsSinceFirst	−0.000016	<0.001
YearsSinceFirst:research_group[T.AD]	−0.000047	<0.001

**Table 2:** Longitudinal mixed-effects Model 1: CTRED predictor.

Predictor	Coef.	*p*-Value
z_prev_CTRED	−0.000015	<0.001
YearsSinceFirsrt	−0.000018	<0.001
YearsSinceFirst:research_group[T.AD]	−0.000110	<0.001
research_group[T.AD]	−0.000457	<0.001

**Table 3:** Longitudinal mixed-effects Model 2: MAPres predictor.

Predictor	Coef.	*p*-Value
z_prev_CTRED	−0.000003	<0.393
YearsSinceFirst	−0.000015	<0.001
YearsSinceFirst:research_group	−0.000052	<0.001
Research_group[T.AD]	−0.000528	<0.001

**Table 4:** Demographic characteristics of the study participants.

Mean ± Std Dev	Cognitively Normal (CN) (1114 Subjects)	Alzheimer’s Disease (AD) (174 Subjects)
Years of Age	75.85 ± 4.66	73.94 ± 7.21
MMSE score	28.38 ± 2.75	20.99 ± 5.22
Females	569 (51%)	105 (60%)
Males	545 (49%)	69 (40%)

## Data Availability

Publicly available datasets were analyzed in this study. This data is available from the Alzheimer’s Disease Neuroimaging Initiative (ADNI) at http://adni.loni.usc.edu.

## Abbreviations

The following abbreviations are used in this manuscript:

AD	Alzheimer’s Disease
CN	Cognitively Normal
MDPI	Multidisciplinary Digital Publishing Institute
MAPres	Mean Arterial Pressure
CSF	Cerebrospinal Fluid
CTRED	Erythrocyte Count in CSF
BBB	Blood Brain Barrier
